# Adequacy of measures of informed consent in medical practice: A systematic review

**DOI:** 10.1371/journal.pone.0251485

**Published:** 2021-05-27

**Authors:** Kerry A. Sherman, Christopher Jon Kilby, Melissa Pehlivan, Brittany Smith

**Affiliations:** 1 Centre for Emotional Health, Department of Psychology, Macquarie University, Sydney, Australia; 2 The Cairnmillar Institute, Melbourne, Australia; University of Bologna, ITALY

## Abstract

As a critical component of medical practice, it is alarming that patient informed consent does not always reflect (1) adequate information provision, (2) comprehension of provided information, and (3) a voluntary decision. Consequences of poor informed consent include low patient satisfaction, compromised treatment adherence, and litigation against medical practitioners. To ensure a well-informed, well-comprehended, and voluntary consent process, the objective and replicable measurement of these domains via psychometrically sound self-report measures is critical. This systematic review aimed to evaluate the adequacy of existing measures in terms of the extent to which they assess the three domains of informed consent, are psychometrically sound and acceptable for use by patients. Extensive searching of multiple databases (PsychINFO, PubMed, Sociological Abstracts, CINAHL, AMED) yielded 10,000 potential studies, with 16 relevant scales identified. No existing scale was found to measure all three consent domains, with most only narrowly assessing aspects of any one domain. Information provision was the most frequently assessed domain, followed by comprehension, and then voluntariness. None of the identified scales were found to have adequate evidence for either high quality psychometric properties or patient user acceptability. No existing scale is fit for purpose in comprehensively assessing all domains of informed consent. In the absence of any existing measure meeting the necessary criteria relating to information, comprehension and voluntariness, there is an urgent need for a new measure of medical consent to be developed that is psychometrically sound, spans all three domains and is acceptable to patients and clinicians alike. These findings provide the impetus and justification for the redesign of the informed consent process, with the aim to provide a robust, reliable and replicable process that will in turn improve the quality of the patient experience and care provided.

## Introduction

Obtaining patient consent is a necessary and critical process enshrined in medical practice, that is characterised within a model of three domains: (provision of) Information, Comprehension (of information by the patient), and Voluntariness (of the patient’s decision without coercion) [1]. The complexity and unfamiliarity of many medical decisions renders decision making challenging for patients with many rarely involved in a shared decision-making process [2, 3]; yet health professionals do not necessarily have a good grasp of the consent process and patient decision-making capacity [4]. Although aspects of consent may be fulfilled by patients completing a consent form [5, 6], research indicates the informed consent process is poorly executed, with widespread reporting of ill-informed patients who agree to procedures for which they have little comprehension [7], suggesting that the provision of consent does not always reflect a well-informed, well-considered, ethical process [5]. With shared decision making the gold standard for medical practice [8–10], it is critical that the medical consent process reflects the patient’s values and goals, and not simply the clinician’s view [11]. There are costly consequences of patients being inadequately informed about medical procedures ranging from low patient satisfaction with care, increased patient regret, poor adherence to treatment plans, both underuse and overuse of the health system [2, 12–17], and patient litigation against medical practitioners [18]. These failures are due in part to the lack of any standardised approach to measuring the consent process [9], and there is a pressing need for standardisation across medical practice contexts [19]. Reviews of the informed consent process for participation in medical research and clinical trials similarly highlight serious shortcomings stemming from lack of standardisation of measures [20–22]. To ensure that truly informed consent is achieved, it is critical that the patient perspective is reflected by assessing the three consent domains using patient-reported measures undertaken in an objective and replicable manner (i.e., using psychometrically-sound measures). Yet, no such systematic investigation has been undertaken to evaluate the quality of current measures available to assess the informed consent process in medical practice, or whether current measures are fit-for-purpose in assessing the three consent domains.

Adequately assessing the extent to which the three domains of consent are being addressed requires clarification of the breadth of these constructs. Providing information, the first critical step in the consent process, entails describing the procedure, anticipated risks/benefits, and available alternatives [3], as well as facilitating the patient to ask questions beyond that which has been provided in the consent documentation [23]. However, there is frequently a mismatch between information given by health professionals and that desired by patients [24, 25].

Closely related to the need for appropriate provision of information is the requirement for the patient to comprehend the information, including their ability to apply the information to their own situation, using this to support their decision [26]. The temporal aspect of comprehension is also important to assess in terms of whether a patient has had sufficient time to make a decision [2, 21, 27]. Comprehension further entails consideration of whether the individual has the cognitive capacity to fully process and understand the information provided [19, 28, 29], suggesting the need to evaluate consent capacity [30] and tailor consent information that accommodates for differing cognitive abilities (e.g., graphical and simplified text-based formats) [31, 32]. There is documented widespread lack of comprehension [22], particularly regarding patient understanding of benefits/risks of surgery and anaesthesia, and alternative treatment options [5, 32–34]. Clearly, any assessment of medical consent should comprehensively capture both the extent to which a patient is informed, as well as the different dimension reflecting their comprehension.

Other factors that may influence informed consent decision-making include cultural or language barriers, and fear of the healthcare system, that is frequently present in minority populations [e.g., 28, 35, 36]. This relates to the third domain of voluntariness, that stipulates the need for informed consent to be undertaken without any duress or undue influence from medical practitioners. Of note, however, is research highlighting that the level of independence in medical decision making differs from person to person, with some individuals requiring greater assistance in arriving at a voluntary decision, whereas others require more time to think and consider the decision on their own. Another critical aspect of voluntariness is the assessment of comprehension and information provision independent of the clinician through patient self-reported measures, whose direct questions through clinical interview may introduce bias by influencing responder’s answers, negating the voluntariness of the decision [19].

An objective and replicable approach is needed to measure the extent to which consent processes are meeting the goals of the three domains of Information, Comprehension, and Voluntariness. Prior systematic reviews have not directly addressed this issue, but relatedly, have highlighted a paucity of reliable and valid scales to assess specific aspects involved in medical decision-making more generally (e.g., extent of patient involvement in decision making [9], patient need for information [25], and participation in clinical trials [20]). To that end, a detailed examination of the measures available to assess the consent process in medical practice is warranted. This study aimed to undertake a systematic review to identify and critically evaluate existing evidence-based measures of patient informed medical consent. Specifically, we aimed to evaluate the adequacy of existing measures in terms of the extent to which they assess the three domains of informed consent and whether they are psychometrically sound, reliable and valid measures. This review paper will make recommendations for the potential application or modification of relevant existing measures and identify gaps in currently available measures for assessing medical consent, in order to provide a robust, reliable and replicable process.

## Methods

This systematic review was conducted in accordance with the PRISMA guidelines (see S1 Checklist for PRISMA checklist).

### Search strategy

Six online journal databases (PsychINFO, PubMed, Sociological Abstracts, CINAHL, AMED) were searched for relevant articles in March 2020 using the following detailed search strategy: (consent* OR withdraw*) OR ((decision* AND (making OR capac* OR conflict* OR regret* OR need* OR support*)) OR competence OR risk* OR coer* OR "mental capac*") OR (underst* OR comprehen* OR benefit* OR harm* OR literacy OR know* OR aware* OR (satisfac* AND (patient OR client)) OR disclos* OR (treat* AND (option* OR alternati*))) OR (autono* OR agen* OR self-determin* OR value* OR choice) OR ("physician-patient relations" OR communication OR dissemination OR "privilege* communic*" OR care OR services OR treatment OR privacy) OR (clinical AND trial OR randomi?ed AND control?ed AND trial OR rct) AND (psychomet* OR valida* OR measure* OR scal* OR survey* OR questionnaire* OR (construction AND (test OR scale))). Reference lists of identified articles were hand searched to ensure all potential studies were captured. The review protocol is not published elsewhere.

### Inclusion and exclusion criteria

The review inclusion and exclusion criteria are listed in Table 1. Wherever the abstract or title indicated exclusion criteria were met, the study was excluded. Full text articles were reviewed when it was unclear if inclusion criteria were met from the title or abstract alone and discussed amongst the research team; papers that met all the inclusion criteria and which did not meet any of the exclusion criteria were included for review.

**Table 1:** Study inclusion and exclusion criteria.

Criterion	Included	Excluded
Type of study	Original study reporting on scale development/validation	Review
	Quantitative	Qualitative
Type of scale delivery	Self-report	Clinical interviews
Type of scale	Paper-based	Other
	Online	
Population studied	Legal age and sound mind to consent	Other
Type of literature	Published peer-reviewed	Conferences, theses, other gray literature
Type of construct	Related to decision making or the consent process	All other constructs
Language	English	Other

### Scale evaluation scoring system

The authors reviewed the included studies for evidence of scale validation. Scale psychometric properties were rated using a previously published scoring system [37], see Table 2. A score of “1” was given when the validation sample exceeded 300, and “0.5” for sample sizes >200 and <300 [38]. For every reported adequate psychometric property (e.g., construct validity) [39], studies received a score of “1” or “0.5” (depending on the scoring of each psychometric property) [40]. When authors did not examine a type of validation or the validation failed, it was scored “0”. Scores for each psychometric property were summed to give an overall score (possible range: 0–16). Ratings were undertaken by two of the authors (MP, BS) with any disagreements discussed until agreement was reached in collaboration with the other authors (KAS, CJK).

**Table 2:** Psychometric properties of scales and rules for evaluation.

Psychometric property	Definition	Method of measurement	Psychometric evaluation criteria	Scoring
Sample				
Validation sample size	Size of the sample used to validate the scale	Sample size (N)	≥200	“0.5” if the sample size was between 200–299 and “1” if the sample was ≥ 300, as this is recommended for scale validation.
Reliability				
Internal consistency	Extent to which all scale items are measuring the same construct	Cronbach’s α	0.70–0.90	“1”: ≥0.7
				“0”: <0.7
	Item total correlation	≥0.40	≥0.40 [37]	“1”: ≥0.40
				“0”: <0.40
Test–retest reliability	Degree of consistency in scores by the same people at two times, assume no construct change	Correlation coefficient between scores from two occasions some time apart (maximum 2–4 weeks)	≥0.70	“1”: ≥0.7
				“0”: <0.7
Validity				
Content	Extent to which items in a scale cover the construct adequately	Based on theory, prior scale devt or lit review, quali feedback from experts, and participant feedback	N/A	“1”: for each instance of evidence that the measure is based on theory/ literature review, or examined by experts in the field, or examined by patients/participants. (Maximum score of 3)
Criterion	How well the scale relates to the “true value" or a “gold standard” for measuring the construct	Correlation coefficient between survey and criterion scores	Higher the correlation the more valid the scale. Acceptable values: significant moderate (*r* > 0.30) to high (*r* > 0.50) correlations	“0.5” if 0.30 < *r* < 0.49
				“1” if *r* >.50 of correlations between the scale and the “gold standard” measure either taken at the same time (*Concurrent*) or in the future (*Predictive*)
				(Maximum score of 2)
Validity				
Construct	Extent to which hypothesized relationships with similar (*Convergent*) or different (*Discriminant*) constructs are confirmed	Correlation coefficient between survey scores and hypothesized variables	Moderate correlations with similar constructs ≥0.3 (*Convergent*) and low correlations <0.30 with different constructs (*Discriminant*)	“1”: evidence of Convergent validity
				“1”: evidence of Discriminant validity (Maximum score of 2)
			Known groups comparisons	“1”: Significant difference in scores between known groups [37]
			Factor analysis	“1”: Factor analysis (either confirmatory or exploratory) confirms hypothesized structure [37] (Maximum score of 2)
Responsiveness to change				
	Sensitivity to change over time	No widely accepted method of measuring responsiveness to change exists [e.g., ANOVA comparing scores over period where change is hypothesized to have occurred; correlation with people’s perceptions of change; 40].	Significant differences in scores over time when the change in consent beliefs are hypothesized to have occurred (e.g., due to information provision/discussion); significant correlation between scale scores’ and respondent’s or other professionals’ perceptions of change.	“1”: Evidence of sensitivity to change over time
		Guyatt Responsiveness Statistic: the ability of the scale to capture clinically meaningful change.	≥0.2: acceptable responsivity	
			>1.00: high responsivity [35]	
Acceptability				
	User perception of ease of use	Self-reported patient user ratings of using the scale.		“1”: Information given on patient perspective of scale

Abbreviations: devt, development; lit review, literature review; quali, qualitative; N/A, Not Applicable.

## Results

**Fig 1:**
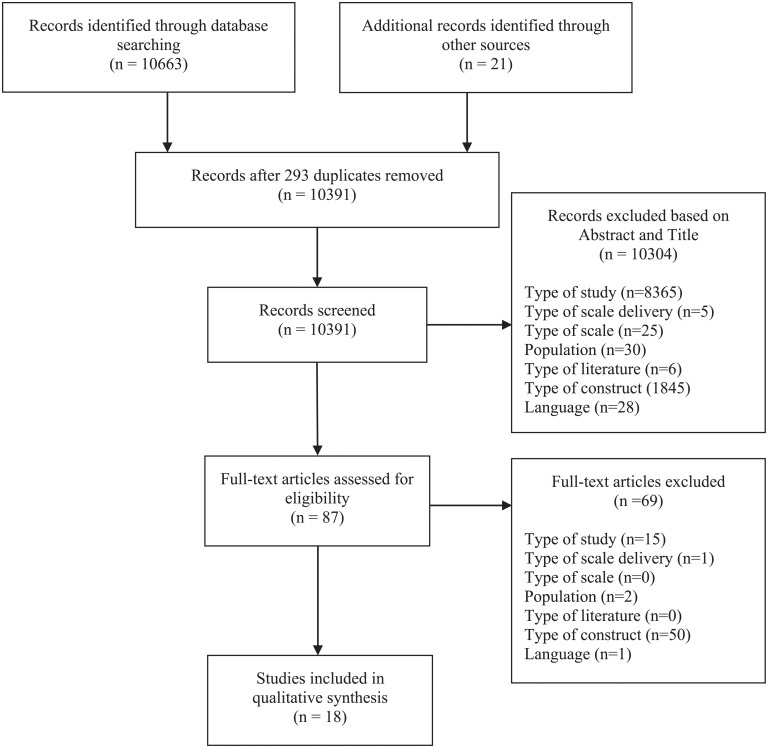
PRISMA diagram.

The results of the literature search are presented in the PRISMA diagram (Fig 1). From 10,663 articles initially identified, 10,370 were unique results (*n* = 293 removed), and after screening for titles and abstracts, a further 10,304 articles were excluded. Of the 66 remaining articles, 8 met inclusion criteria. Hand-searching of reference lists of these included articles yielded a further 8 papers that met inclusion criteria, resulting in 16 included articles (representing 19 different studies reporting on 16 different scales).

The description and evaluation of reviewed scales is provided in Tables 3 and 4. The total sample size across all 19 studies was 8195 participants (range *N* = 45 to 2351), with nine scales meeting the criterion of having adequate validation study sample size [41–49]. Scale length ranged from 5 [50, 51] to 36 items [46]. Studies were based in the United States (*n* = 8), Canada (*n* = 2), United Kingdom (*n* = 2), France (*n* = 2), Germany (*n* = 1) and Netherlands (*n* = 1).

**Table 3:** Summary of scale papers included for review.

Scale Name	Construct/s measured	Example item	Higher score interpretation	Subscales related to domains of consent		
(No. items)	(Subscales)	Range of scores		Information	Comprehension	Voluntariness
Combined Outcome Measure for Risk communication and treatment Decision making Effectiveness (COMRADE, 20 items; 41)	Risk communication (Risk communication) Decision-making effectiveness (Confidence in decision)	“The doctor gave me enough information about the treatment choices available.” (NR)	Greater risk communication and confidence in treatment decision, respectively.	✔ (Risk communication)		✔ (Confidence in decision)
Doctor-patient communication questionnaire [13 items; 50]	Doctor-patient communication: listening, confidence, empathy, decision-making, quality of care, information and reassurance (Communication)	“Did the doctor explain the advantages and disadvantages of the treatment or care strategy?” (1 = no to 4 = yes).	Greater doctor-patient communication	✔ (Communication)		
Generic Medical Interview Satisfaction Scale [G-MISS, 16 items; 42]	Patient experiences and satisfaction with medical consultations (Sense of post-communication relief) (Communication) (Compliance)	“The doctor told me all I wanted to know about my illness” (1 = strongly disagree to 5 = strongly agree).	(1) Greater alleviation of illness-related distress, (2) Greater communication comfort between the patient and doctor (3) Greater intent to follow doctor’s instructions.	✔ (Sense of post-communication relief)		
Facilitation of Patient Involvement Scale [9 items; 43]	Patients’ perceptions of physician’s facilitation	“My doctor explains all treatment options to me so that I can make an informed choice” (1 = none of the time to 6 = all of the time).	Greater perception of patient facilitation in healthcare.	✔ Patients’ perceptions of physician’s facilitation		
Brief Health Literacy Screen [5 items; 54]	Health Literacy	“How often do you have trouble understanding what your doctor, nurse, or pharmacist (druggist) tells you about your health or about treatments?” (1 = always to 5 = never).	Higher level of health literacy		✔ Health literacy	
Perceived Efficacy in Patient-Physician Interactions Questionnaire [PEPPI, 10 items and PEPPI-Short, 5 items; 51]	Patient- perceived self-efficacy: the subjective sense of patients’ confidence when interacting with their physicians.	“How confident are you in your ability to understand what a doctor tells you?” (1 = not at all confident to 5 = very confident).	Higher patient self-efficacy	✔ Perceived efficacy in patient-physician interactions		
Healthcare Relationship Trust Scale [15 items; 52]	Collaborative trust: (a) Knowledge Sharing; (b) Emotional Connection; (c) Professional Connection; (d) Respect; (e) Honesty; and (f) Partnership. (Interpersonal communication) (Respectful communication) (Professional Partnering)	“Discusses options and choice” (0 = none of the time to 4 = all of the time).	Patient trust in healthcare relationships	✔ (Interpersonal connection) (Professional partnering)		
Subjective Tests of Health Literacy and Numeracy [22 items; 53]	Health Literacy and Numeracy (Functional health literacy) (Communicative health literacy) (Critical health literacy) (Perceptions of quantitative ability)	“Health literacy: Since being diagnosed with diabetes have you extracted information you wanted? Numeracy: How good are you at working with fractions?” Health literacy subscales: (1 = never to 4 = often); numeracy scores rating scale from (1 to 6).	Higher health literacy and subjective rating of numeracy abilities and preferences.	✔ (Communication health literacy)	✔ (Functional health literacy) (Critical health literacy)	
Decisional Conflict Scale [16 items; 44]	Decisional conflict: uncertainty, informed, values clarity, support, effective decision (Uncertainty) (Effective decision-making) (Factors contributing to uncertainty)	“I feel I have made an informed choice” (1 = Strongly agree to 5 = Strongly disagree).	Higher decisional conflict		✔ (Factors contributing to uncertainty)	
DelibeRATE [9 items; 55]	Deliberation process	“I know enough about each option to help me decide” (1 = Strongly disagree to 7 = Strongly agree).	The more ready individuals were to make a decision about which surgery to choose		✔ Deliberation process	
The Autonomy Preference Index [API, 23 items^;^ 45]	Decision-making (Decision-making preference) Information-seeking (Information-seeking preference)	“You should go along with your doctor’s advice even if you disagree with it.” (1 = Strongly disagree to 5 = Strongly agree).	Strongest preferences in favor of decision making or information seeking	✔ (Information-seeking)		✔ (Decision-making preference)
University of California, San Diego Brief Assessment of Capacity to Consent [UBACC, 10-items; 56]	(1) Understanding, (2) Appreciation, (3) Reasoning	Is it possible that being in this study will not have any benefit to you? (0 = Incapable to 2 = Capable).	Clear capability to give consent		✔ Understanding Appreciation Reasoning	
Decision Evaluation Scales [36-items; 46]	Uncertainty about and satisfaction with the decision, informed choice, effective decision making, responsibility for the decision, perceived riskiness of the choice, and social support regarding the decision (Satisfaction–Uncertainty) (Informed Choice) (Decision Control)	“I feel pressure from others in making this decision” (1 = Strongly disagree to 5 = Strongly agree).	Greater: (1) satisfaction (2) informed choice (3) decisional control		✔ (Informed choice)	✔ (Satisfaction-uncertainty) (Decision-control)
The Health Care Empowerment Questionnaire [HCEQ, 10-items; 47]	Feeling of control, interaction with health professionals, and decisional process (Involvement in decisions) (Degree of control) (Involvement in interactions)	“That you obtain all the information you want” (1 = Not at all to 4 = Extremely).	Greater individual empowerment in relation to personal health care and services.	✔ (Involvement in decisions) (Involvement in interactions)		✔ (Degree of control)
Preparation for Decision Making scale [10-items^;^ 48]	Perception of how useful a decision aid or other decision support intervention is in preparing the patient to communicate with their practitioner at a consultation and to make a health decision. (Preparation for decision-making)	“Help you think about which pros and cons are most important?” (1 = Not at all to 5 = A great deal).	Patients are highly prepared for decision-making		✔ (Preparation for decision-making)	
9-item Shared Decision Making Questionnaire [SDM-Q-9, 9-items; 49]	Shared decision-making	“My doctor precisely explained the advantages and disadvantages of the treatment options” (0 = Completely disagree to 5 = Completely agree)	Greater shared decision-making			✔ Shared decision-making

Abbreviations: No., Number; NR, No Response.

Note: Higher score interpretation indicates what a higher score on a given scale means (e.g., higher or lower presence of specified construct).

**Table 4:** Evaluation of scale quality.

Scale	Sample	Reliability		Content Validity	Crit Validity		Constru validity		FA		SA	Acceptability	Qual Score /16 (%)
		IC	TRR		Con	Pre/KG	Conv	Div	E	C			
COMRADE [41]	n = 715	α = .92	-	T/LR, EF, UF	✔[29]	-	✔	-	✔	-	-	80.76% completion.	
Score	1	1	0	3	1	0	1	0	1	0	0	0	8 (50)
Doctor-patient communication questionnaire [50]	n = 156	α = .89	-	T/LR, EF, UF	-	✔	-	-	-	✔	-	88% completion.	
Score	0	1	0	3	0	0.5	0	0	0	1	0	0	5.5 (34.38)
G-MISS [42]	n = 1822	α = .85	-	T/LR, UF	-	-	-	-	✔	-	-	5.2% of patients with missing values >20%.	
Score	1	1	0	2	0	0	0	0	1	0	0	0	5 (31.25)
Facilitation of Patient Involvement Scale [43]	n = 1035	α≥.89	r≥.85 (8 to 10 weeks)	EF	-	✔	✔	-	✔	-	-	-	
Score	1	1	1	1	0	1	1	0	1	0	0	0	7 (43.75)
Brief Health Literacy Screen [54]	n = 100	α = .79	-	T/LR, UF	✔	✔	-	-	✔	-	-	No reported discomfort in completion.	
Score	0	1	0	2	1	1	0	0	1	0	0	1	7 (43.75)
PEPPI & PEPPI (Short) [51]	n = 163	α≥90^a^ α≥.82^b^	-	EF, UF	-	-	✔	✔	✔	-	-	-	
Score	0	1	0	2	0	0	1	1	1	0	0	0	6 (37.50)
Healthcare Relationship Trust Scale [52]	n = 99	α≥.92	r = .59 (2 to 4 weeks)	EF, UF	-	-	✔	-	✔	-	-	-	
Score	0	1	0	2	0	0	1	0	1	0	0	0	5 (31.25)
Subjective Tests of Health Literacy and Numeracy [53]	n = 102	α = .83	-	T/LR	✔^c^	✔	-	-	-	✔	-	-	
Score	0	1	0	1	0.5	1	0	0	0	1	0	0	4.5 (28.13)
Decisional Conflict Scale [44]	n_1_ = 151^d^ n_2_ = 115^e^ n_3_ = 283^f^ n_4_ = 360^g^	α≥0.78	r≥.78 (2 weeks)	T/LR	-	✔	✔	-	-	-	delay vs make decision	-	
Score	1	1	1	1	0	1	1	0	0	0	1	0	7 (43.75)
DelibeRATE [55]	n = 54	α≥.95	-	T/LR	-	✔	-	-	-	-	Pre vs post delib scores	-	
Score	0	1	0	1	0	0.5	0	0	0	0	1	0	3.5 (21.88)
API [45]	n = 312	α = .82	r≥.82 (two weeks)	EF, UF	✔	-	-	-	✔	-	-	-	
Score	1	1	1	2	1	0	0	0	1	0	0	0	7 (43.75)
UBACC [56]	n = 157	α≥.76	-	T/LR, EF	✔^c^^h^	-	-	-	✔	-	-	-	
Score	0	1	0	2	0.5	0	0	0	1	0	0	0	4.5 (28.13)
Decision evaluation scales [46]	n = 343	α≥.75	-	EF	-	✔	✔	-	✔	-	-	87% completion.	
Score	1	1	0	1	0	1	1	0	1	0	0	0	6 (37.5)
HCEQ [47]	n = 873	α = .83	-	T/LR, EF, UF	-	-	-	-	✔	✔	-	-	
Score	1	1	0	3	0	0	0	0	1	1	0	0	7 (43.75)
Preparation for Decision Making scale [48]	n = 400	α≥.92	-	T/LR	-	✔	0	-	✔	-	-	-	
Score	1	1	0	1	0	1	-	0	1	0	0	0	5 (31.25)
SDM-Q-9 [49]	n = 2351	α≥.90	-	T/LR	-	-	-	-	✔	-	-	Response rates <80%.	
Score	1	1	0	1	0	0	0	0	1	0	0	0	4 (25)
Number of scales meeting specific criterion/16 (%)	9 (56.25)	16 (100)	3 (18.75)	3 (18.75)	3 (18.75)	6 (37.5)	6 (37.5)	1 (6.25)	12 (75)	3 (18.75)	2 (12.5)	1(6.25)	

Abbreviations: Crit, Criterion; Constru, Construct; FA, Factor Analysis; SA, Sensitivity Analysis; Qual, Quality; IC, Internal consistency; TRR, Test-retest reliability; Con, Concurrent. Pre, Predictive; KG, Known Groups; Conv, Convergent; Div, Divergent; E, Exploratory; C, Confirmatory; T/LR, Theory/Literature review; EF, Expert feedback; UF, User feedback; delib, deliberation.

^a^Internal consistency for the PEPPI.

^b^Internal consistency for the PEPPI (Short).

^c^Spearman Rank coefficient reported.

^d^Health science students.

^e^Health employees.

^f^Cardiac/respiratory patients.

^g^Breast cancer patients.

^h^Analysis conducted at item level.

### Assessing domains of consent

Information provision was assessed by 9 (56%) scales:[41–43, 45, 47, 50–53]. Comprehension of information was also assessed by 7 (44%) scales: [44, 46, 48, 53–56]. Voluntariness was assessed by 5 scales (31%): [41, 45–47, 49]. None of the scales assessed all three domains of consent. Six (37.5%) scales assessed two consent domains: [41, 42, 45–47, 53]. The remaining 10 (62.5%) scales assessed only one domain of consent.

### Scale quality

#### Reliability

None of the scales were awarded a full score (i.e., 3) for reliability. Three scales (19%) provided evidence of both internal consistency and temporal stability: [43–45].

#### Validity

None of the scales were awarded a full score (i.e., 10) for evidence supporting their validity. Of those with the greatest evidence for validity, COMRADE [41] scored 6/10 and [47, 51, 54] each scored 5/10.

#### Acceptability to users

Only one scale provided information from the patient perspective on acceptability of the scale, specifically regarding comfort in responding to the questions (Brief Health Literacy) [54]. Information about completion rates was provided by 5 (38%) scales: [41, 42, 46, 49, 50].

#### Overall scores

Total quality assessment scores out of 16 ranged from a low of 3.5 to a high of 8. The COMRADE [41] had the highest overall score (8/16) closely followed by: [43–45, 47, 54] all scoring 7/16. The Decision Evaluation Scale [46] and PEPPI and PEPPI-Short [51] were not far behind, both scoring 6/16. The most frequently met quality criteria were internal consistency reliability (*n* = 16, 100%), exploratory factor analysis (*n* = 12, 75%), and adequate validation sample size (*n* = 9, 56%). Divergent validity (*n* = 1, 6.25%) and sensitivity analysis (*n* = 2, 12.5%) were the most poorly met quality criteria, followed by concurrent and content validity, test-retest reliability and confirmatory factor analysis (all *n* = 3, 18.75%).

## Discussion

This systematic review identified and evaluated existing approaches to measuring the adequacy of the informed consent process in medical practice. After an extensive literature search of more than 10,000 potential studies, only 16 scales were identified that assess any of the three domains of informed consent. Most of the identified scales have extensive limitations. In terms of the content that is being assessed, no existing scales measure all three consent domains. Several scales measure two of the three domains [41, 42, 45–47, 53]. Moreover, the exact aspect of each domain tapped into by these measures were quite different. Hence, in terms of content being assessed, no one scale is fit for purpose in comprehensively assessing all domains of informed consent, and at best several measures would need to be used in conjunction to assess all domains comprehensively.

Of the three top ranking scales assessing the Information domain, the COMRADE [41] focuses solely on whether risk-related information has been provided for the patient, and the Facilitation of Patient Involvement Scale [43] and the HCEQ [47] capture the extent to which the patient perceives that the physician has facilitated a shared decision. Of the two latter scales, the Facilitation of Patient Involvement Scale [43] has more extensive evidence for validity (criterion and construct) having demonstrated that it can predict patient satisfaction following medical procedures and demonstrates logical relationships with similar constructs. As such, a combination of components from the COMRADE [41] and the Facilitation of Patient Involvement Scale [43] may be appropriate to comprehensively assess the Information domain.

The three highest scoring scales for the domain of Comprehension [44, 46, 54] have similar levels of evidence for their validity, with one scale [44] additionally providing evidence for stability over time. The Decisional Conflict Scale [44] captures extent of uncertainty around decision making, whereas the Brief Health Literacy Screen [54] assesses self-perceptions of comprehending the information provided by a physician. More directly related to the informed consent process is the informed choice subscale of the Decision Evaluation Scales [46], assessing self-perceptions of being adequately informed about a procedure in order to make a choice. Perhaps due to the inherent difficulty in developing measures that assess context-specific knowledge (e.g., details of a surgical procedure), none of these scales assess accuracy of knowledge. What is missing from any of these higher rated scales is consideration of whether the patient is able to apply the information provided to their own situation, using this to support their decision making [26], and the temporal aspect of the consenting process [2, 27], two factors that are key to comprehension.

For the Voluntariness domain, the COMRADE [41] scored highest with a subscale that assesses patient confidence to make a decision, followed by the API [45] and HCEQ [47] both of which assess the patient’s preferred role or amount of control in the decision-making process. The API [45] has greater evidence for reliability and validity, compared with the HCEQ [47], potentially making it the more useful measure. Although tapping into aspects of patient confidence and patient preferred role in this decision making, an aspect neglected by these higher rated scales is whether the consent (decision) is being made free of undue influence or duress from the medical team, the main caveat of voluntariness [19].

In assessing the quality of the existing scales reflecting aspects of the informed consent process, it is important to consider how the scale may be implemented in clinical practice in terms of: (1) does the scale have high quality psychometric properties to ensure that the variability in scores on the scale truly reflect variability in the underlying construct (e.g., comprehension), rather than measurement error?; (2) does the scale preferably measure more than one domain of informed consent, to minimize patient burden and have the most parsimonious measurement approach?; and, (3) practical considerations regarding user (patient) experience of using the scale, and the administration, scoring and interpretation of the scale. For psychometric quality, the COMRADE [41] scored highest, followed by [43–45, 47, 54], with three of these scales assessing more than one domain [41, 45, 47]. Information is the most frequently assessed domain of these higher quality scales, yet, the COMRADE assesses a very narrow perspective (i.e., whether risk-related information has been provided), neglecting crucial aspects including whether the procedure and available alternatives are adequately described [3] and if the patient was able to ask questions prior to consenting [23]. Hence, as a measure of the Information domain, COMRADE alone is not fit for purpose and needs to be supplemented. In the interests of providing a parsimonious approach to measuring informed consent, the COMRADE does provide a measure of Voluntariness, although this alone is not fit for purpose as it neglects to assess whether consent (the decision) is being made free of undue influence or duress from the medical team [19]. Moreover, since the COMRADE neglects to assess comprehension, other high scoring measures in this domain (e.g., Brief Health Literacy Screen [54]) may need to be invoked.

Importantly, in reviewing the 16 scales assessing aspects of the consent process, only the San Diego Brief Assessment of Capacity to Consent [56] was purposely designed for measuring the consent process, specifically in randomized controlled trial research. However, this scale has yet to be subjected to rigorous validity assessment. All other measures were designed to assess specific aspects of communication or decision making relating to medical procedures but were not purpose built to comprehensively measure any one of the domains of consent, calling into question the adequacy of even a combination of these measures to assess each domain.

The results of this review should be considered with some key limitations in mind. As is the case with any review, the adopted search strategy may not have identified all relevant articles. Yet, our rigorous broad search strategy and extensive manual review of the large volume of initial papers support the notion that the results of this review are representative of the field. Further, the review did not look at the consent process for individuals who cannot give consent for themselves (e.g., issues of soundness of mind or legalities in consent such as in minors). In addition, this review did not consider the impact of culture on medical consent, which may influence the level of voluntariness for example, and thus, culture-specific scales may be needed in certain collectivistic countries to adequately assess medical consent. These were issues beyond the scope of this review and were therefore not included. However, future work in the medical consent field may benefit from a similar review as this one on these more complex situations. Lastly, this review did not include qualitative studies of medical consent scales and hence did not allow for a more in-depth analysis of the patients’ subjective experience with the scales; it is recommended that future research elicit patients’ responses to open-ended questions about their experiences with medical consent scales.

## Conclusion

This review has highlighted the complex nature of consent reflected within the three domains of information, comprehension and voluntariness, and the need to ensure that these multiple aspects are reflected in both measurement and practice. Of greatest concern is the absence of any established, standardized, reliable and valid measure of consent addressing these three domains, suggesting serious limitations in the current approaches to the consenting process. In summary, as a starting point, the COMRADE is the most likely contender for assessing certain aspects of informed consent. Yet, it only provides a modest level of coverage of the informed consent domains, and it lacks clear evidence for validity and user acceptability—something inherently lacking in most of the reviewed scales. Clearly, what is needed is not only a psychometrically high-quality measure, but also one that is easy for patients to use and has a high degree of perceived relevance to patients and clinicians alike. Unfortunately, only one of the higher scoring scales reviewed, the Brief Health Literacy Screen, assessed the usability of the scale, yet this scale only covers one of the elements of informed consent (Comprehensiveness). Therefore, there is need for a new measure of medical consent to be developed that has robust psychometric properties, spans all three domains and is acceptable to patients and clinicians alike, that will in turn improve the quality of the patient experience and care provided.

## Supporting information

S1 Checklist PRISMA checklist. (DOC)
